# Effects of digital skills and other individual factors on retirement decision-making and their gender differences

**DOI:** 10.1007/s10433-023-00784-9

**Published:** 2023-10-07

**Authors:** Martin Lakomý

**Affiliations:** Faculty of Business and Economics, Mendel University in Brno, Brno, Czech Republic

**Keywords:** Retirement intentions, Older workers, Digital skills, Family structure, Panel analysis, SHARE

## Abstract

Increasing the pension age as a dominant solution to population ageing does not bring desirable outcomes, if not accompanied by other essential measures in lifelong learning and fighting age discrimination. Moreover, rapid digitalisation and automation in the labour market bring additional uncertainties for the growing group of older workers. The analysis is based on the SHARE data from Waves 5, 6, and 7 and examines predictors of retirement intentions by two different estimation methods. While digital skills are positively associated with a willingness to stay in the labour market in the random-effect modelling, fixed-effects regression shows no correlation between digital skills and retirement intentions. This difference means that digital skills do not correlate with retirement intentions once we control for time-invariant individual characteristics. Thus, increasing ICT literacy among older workers can have a very limited potential for extending working lives. In contrast to this result, starting to be self-employed, health improvement, having an additional grandchild, and losing a partner increase the willingness to work longer. The study identifies the factors shaping retirement intentions, which should be reflected in any effective social policy.

**Supplementary Information:**

The online version contains supplementary material available at 10.1007/s10433-023-00784-9.

## Introduction

The present paper is concerned with predictors of staying longer in the labour market as population ageing leads to an older workforce and a lower proportion of people in the so-called productive age. These processes are pronounced in Europe, a front-runner in demographic changes (Sobotka 2008; Timonen 2008). While being a considerable success for humankind, population ageing is a challenge to labour markets, welfare systems, and the workforce in each country (Grundy 2008; Maltby et al. 2017). The EU has reacted with active ageing policies based mostly on extending working lives (Walker and Maltby 2012; Krekula and Vickerstaff 2017), which can unintentionally increase inequalities in later life (Timonen 2016; Ní Léime et al. 2017; Lakomý 2019).

The increasing pension age as the dominant solution to population ageing has several stark limitations. Older adults experience discrimination in the labour market (Loretto et al. 2007; Oude Mulders et al. 2014), facing more prevalent long-term unemployment (Hardy et al. 2018; Rašticová et al. 2020) and many stereotypes (Ng and Feldman 2012). They also show less willingness to participate in further training (Ng and Feldman 2012) and have health or family reasons to leave the labour market (Radl 2013; Damman 2016). The situation of older adults—who are heterogeneous in terms of skills and resources—needs to be addressed by proper measures in order to create adequate working opportunities for older adults (Hardy et al. 2018; Ní Léime et al. 2017).

The risk of decreasing employability of older workers is a threat to older adults, employers, and entire societies. First, the income from paid work provides financial resources, interpersonal relations, health, and quality of life in general (Wang and Marcotte 2007; Blix 2017). Second, employers—and the labour market in general—increasingly depend on older workers with their specific competencies and experience, who become even more indispensable due to the ageing workforce (Loretto et al. 2009; Grass and Weber 2017; Matt et al. 2020). Third, the social cohesion and stability of the society depend on the relative prosperity of its members (Degryse 2016; Blix 2017). Hence, older adults at risk of long-term unemployment amid the politics of extended working life pose a threat to the social system as such, not to mention the pressure exerted on economic, social, and health systems.

This paper examines the research question *Which individual factors shape retirement intentions, and how do they differ by gender?* Its empirical contribution is in a) examining several factors not adequately reflected by existing research (digital skills, demand for digital skills, family structure, type of employer), b) showing how these factors work for men and women, c) including a variety of European countries, d) providing robust effects as a supplement to potentially spurious effects. In contrast, this study does not deal with country-specific pension and social policy systems, individual pension eligibility, and actual exit from the labour market; these aspects are better addressed by the existing research based on data more appropriate for these purposes. The paper uses data from three waves of SHARE, comparing a standard regression analysis with models based only on changes within individuals to illustrate the substantial differences based on the analytical approach.

## Literature review

### Predictors of retirement

This study examines several characteristics influencing retirement intentions, grouped into time-invariant characteristics, digital skills, family characteristics, and other time-variant factors. The distinction based on time (in)variance is crucial, as only changeable factors have identifiable effects over time that might be modified (by an individual, employer or state). Most studies below focus on antecedents of retirement timing/planning in the European context.

First, age (in the initial wave), gender, and level of education are invariable characteristics covered by this study. While higher age unsurprisingly relates to actual retirement (Fisher et al. 2016; Dingemans et al. 2017; Axelrad and Mcnamara 2018), studies focusing on older workers have found that retirement intentions decrease with age for workers staying in the labour market (Damman et al. 2011; Hochman and Lewin-Epstein 2013; Pilipiec et al. 2022). The role of gender is complex, with women retiring earlier due to family and household responsibilities (Dingemans et al. 2017; Dudová and Pospíšilová 2022; Pilipiec et al. 2022) or—especially in the Anglo-Saxon context—retiring later because of lower financial security (Finch 2014; Léime 2017). Some studies have presented no gender differences in retirement timing/intentions, arguing that more gendered factors got into balance (Hochman and Lewin-Epstein 2013; Radl 2013; Axelrad and Mcnamara 2018). Not least, higher education is generally associated with later retirement, since it provides better employment opportunities and higher intrinsic motivation (Fisher et al. 2016; Dingemans et al. 2017; Pilipiec et al. 2022). These well-established time-invariant factors are not central to this study, and no hypotheses are formulated for them.

Second, the role of digital skills in retirement decision-making has not been clarified by previous studies. The long-term digitalisation and automation in the labour market were further accelerated by a) rapid technological development, b) labour force deficiency, and c) the Covid-19 pandemic. Older workers are more affected by these processes as this age group generally fares worse in several aspects of digital skills (Komp-Leukkunen et al. 2022). Hence, some groups of older workers can be left behind in precarious jobs or long-term unemployment situations amid the development towards the digital economy (Grass and Weber 2017; Hardy et al. 2018). However, no study seems to examine this factor in connection to retirement intentions, and thus, the expectations of this paper are based on indirect evidence. For instance, Van Yperen and Wortler (2017) and Komp-Leukkunen et al. (2022) claim that digital skills are a precondition for some work types (teleworking, blended working), which can fit the needs of older workers well. Moreover, Lissitsa et al. (2017) illustrate that digital skills in a later career signal competence to potential employers and relate to higher income and employment benefits. Hence, this text assumes that lower digital skills limit opportunities in the labour market, while the opposite causation with decreasing digital skills after entering retirement was already identified (Cavapozzi and Dal Bianco 2022). Digital skills can play a role in both labour supply (people with higher digital skills are more employable) and labour demand (certain employers favour workers with higher digital skills). All hypotheses appear behind the relevant assumptions in the text.

#### H1a

Higher digital skills relate to lower retirement intentions.

#### H1b

Higher demands on digital skills in a job relate to higher retirement intentions.

Third, family characteristics—such as partnership, children, grandchildren, parents and other relatives—and their impact are another under-researched area. The role of family in retirement decision-making can indicate a) a joint decision of partners, b) financial need to work longer in the case of more dependent relatives, or c) retirement because of time constraints emerging from caregiving (Matthews and Fisher 2012). Surprisingly, none of the recent research has indicated the effect of caregiving on retirement (Szinovacz et al. 2001; Forma 2009; Dingemans et al. 2017), except for women caring for a spouse in one study (Dentinger and Clarkberg 2002). The authors of the present paper checked that the provision of care was not associated with retirement plans and used the information on family structure instead (which possibly indicated all three aspects a-c mentioned above). Studies addressing the effect of marital status or partnership have shown that earlier retirement is connected to having a retired partner (Radl 2013; Fisher et al. 2016) or having a partner in general (Dingemans et al. 2017). However, substantial gender differences exist here. For instance, divorced people are prone to work longer, which may indicate a need for additional income among divorced women (Hochman and Lewin-Epstein 2013; Dudová and Pospíšilová 2022). Later retirement is also connected to having children (Dingemans et al. 2017) and grandchildren (Radl 2013; Dingemans et al. 2017), although these factors are not always relevant (Szinovacz et al. 2001; Hochman and Lewin-Epstein 2013).

#### H2a

Having a partner relates to higher retirement intentions.

#### H2b

The number of children does not relate to retirement intentions.

#### H2c

The number of grandchildren does not relate to retirement intentions.

Fourth, other time-variant factors covered by this study are the type of employer, health, and financial situation. The existing studies reflect several characteristics of job (satisfaction, experience, position, industry), which are not consistently associated with the decision to retire (Radl 2013; Fisher et al. 2016; Axelrad and Mcnamara 2018). An exception is the positive effect of being self-employed on extended working (Radl 2013; Wahrendorf et al. 2017); two potential explanations are their lower pension eligibility (Wahrendorf et al. 2017) and/or higher flexibility to adjust the workload (Lain and Vickerstaff 2014). This contribution tests changes in the type of employer, which rather indicates the latter explanation. Regarding the public sector, it could provide less precarious jobs contrasting with higher pensions, while Pilipiec et al. (2022) have not found any difference from the private sector. Then, good health is one of the strongest predictors of working longer (Hochman and Lewin-Epstein 2013; Fisher et al. 2016; Dingemans et al. 2017; Wahrendorf et al. 2017; Axelrad and Mcnamara 2018). Finally, different measurements of financial situation appeared in previous research, showing extended working life for individuals with lower pension (Dingemans et al. 2017), higher income (Pilipiec et al. 2022), inadequate financial resources (Fisher et al. 2016), and lower wealth (Damman et al. 2011). In contrast, neither wealth nor income has an effect in the study of Hochman and Lewin-Epstein (2013) and the literature review of Fisher et al. (2016) has found no consistent answer to this issue. Various findings reflecting the role of the financial situation are based on the assumption that workers aim to have adequate assets before entering retirement; at the same time, lower-income groups have fewer opportunities to work longer (Dingemans et al. 2017; Pilipiec et al. 2022). Additionally, working in a white-collar job reduces retirement intentions (Dudová and Pospíšilová 2022), while a specific industry does not seem to affect it (Pilipiec et al. 2022). This text finds it difficult to cross-nationally compare any types of wages, assets, and potential pensions and explores the effect of the subjective financial situation of the household.

#### H3a

Being self-employed relates to lower retirement intentions.

#### H3b

Worse health relates to higher retirement intentions.

#### H3c

Worse financial situation relates to lower retirement intentions.

### Gender differences

Apart from the overall effect of some characteristics, the paper tests potential gender differences in some of these effects. The analysis focuses on gender differences due to different gender roles (Loretto and Vickerstaff 2013) and the intersection of age and gender inequalities in the labour market (Krekula et al. 2018). Hochman and Lewin-Epstein (2013) used data from 13 European countries to show that having a retired partner increases retirement intentions for women, while no partner decreases these intentions for men. A study of US workers from six companies (Dentinger and Clarkberg 2002) has concluded that caregiving for a spouse connects to earlier female retirement and later male retirement. Further, tertiary education reduces retirement intentions in men, with the coefficients for children, grandchildren, age, income, health, and other characteristics being similar across gender (Hochman and Lewin-Epstein 2013). Finally, Dudová and Pospíšilová (2022) have found that Czech women are not protected from earlier retirement by a white-collar job and secondary education, but evince a stronger beneficial effect of tertiary education. Some studies also conclude that women retire earlier due to higher caregiving obligations (Dudová and Pospíšilová 2022; Pilipiec et al. 2022), but recent empirical evidence for this statement is scarce. The present text builds on the existing knowledge by suggesting the following hypotheses:

#### H4a

The effect of an additional spouse, children, grandchildren, and living parents is stronger for women.

#### H4b

The negative relationship between being self-employed and retirement intentions is stronger for women than men.

#### H4c

The negative relationship between health improvement and retirement intentions is stronger for men.

## Data and methods

### Sample

The analysis is based on data from the Survey of Health, Ageing and Retirement in Europe (SHARE). SHARE is a project that has been interviewing panel samples of 50 + from European countries since 2004. While 29 countries participated in at least one wave of data collection, the number of countries (and respondents for a specific country) varies over time (Börsch-Supan et al. 2013; Börsch-Supan 2017a, b, 2019). This study capitalises on the ICT module interviewed since Wave 5 in 2013. The ITC module was also present in Wave 6 in 2015 (for all respondents), Wave 7 in 2017 (only for respondents present in Wave 3), and Wave 8 in 2019–2020 (only for new respondents). The analysis utilises the panel dimension of the data, keeping only respondents from Waves 5–7 present in at least two waves (unbalanced sample).

The SHARE data contain a self-evaluation of digital skills and demand for digital skills at current work from the ITC module, as well as other information essential for the studied topic (family structure, health, financial situation, type of work, etc.). The final sample is limited to respondents without missing values in these variables (8.6% observations dropped) and respondents working in at least two waves (otherwise, they would not complete the key questions). Furthermore, the sample includes European countries (excluding Israel) and respondents between 50 and 65 years of age in the initial wave of the analysis. This specification produces a final sample of 9126 respondents (and 18,940 observations in time) from 15 European countries described in Table 1. Regarding the differences between subsamples of men and women used in the analysis, women have tertiary education more often, possess excellent digital skills less often, need digital skills slightly more often, work more in the public sector, and more prevalently live without a partner. Otherwise, men are comparable to women (even in their decision to retire).

**Table 1:** Descriptive statistics of the sample of all used observations from three waves. *Source:* These calculations use data from SHARE, Waves 5, 6, and 7

Variable	Categories or range	Total (% or mean)	Men (% or mean)	Women (% or mean)
Decision to retire	Yes	43.9	44.4	43.5
Age	50–70	57.22	57.66	56.82
Level of education	ISCED 0, 1	6.1	6.9	5.4
	ISCED 2–4	57.6	58.9	56.5
	ISCED 5, 6	36.3	34.2	38.1
Digital skills	Never used	5.3	6.0	4.8
	Poor	10.2	10.6	9.7
	Fair	24.4	23.2	25.4
	Good	33.5	31.2	35.6
	Very good	18.0	18.1	17.9
	Excellent	8.6	10.9	6.6
Demand for digital skills at work	Yes	74.2	73.1	75.1
Partner in the household	Yes	80.6	85.1	76.6
Number of living parents	0–2	0.69	0.65	0.73
Number of children	0–10	2.07	2.07	2.06
Number of grandchildren	0–15	1.29	1.15	1.41
Type of employer	Private sector	52.1	56.7	48.0
	Public sector	33.2	23.4	42.0
	Self-employed	14.7	19.9	10.0
Type of job	Low skilled blue collar	11.9	13.2	10.8
	High skilled blue collar	12.6	20.7	5.3
	Low skilled white collar	37.4	24.8	48.6
	High skilled white collar	38.1	41.3	35.3
Health status	Poor or fair	17.8	18.4	17.1
	Good	39.5	38.5	40.5
	Very good or excellent	42.7	43.1	42.4
Financial situation	Poor	23.6	22.8	24.2
	Good	28.4	28.3	28.5
	Very good	48.0	48.9	47.3
Wave	Wave 5	46.5	46.3	46.7
	Wave 6	47.8	47.8	47.7
	Wave 7	5.7	5.9	5.6
Country	Austria	3.9	4.3	3.7
	Belgium	9.4	9.4	9.3
	Czechia	6.6	6.8	6.3
	Denmark	13.0	13.8	12.3
	Estonia	9.2	7.2	11.0
	France	5.5	4.9	6.2
	Germany	12.7	12.8	12.7
	Greece	2.2	2.7	1.9
	Italy	6.7	7.3	6.1
	Luxemburg	2.1	2.0	2.3
	Poland	0.5	0.6	0.3
	Slovenia	3.0	2.9	3.0
	Spain	8.2	8.9	7.6
	Sweden	9.1	8.4	9.7
	Switzerland	7.8	8.0	7.6
*N*—measures in time		18,940	8908	10,032

### Outcome variable

The *decision to retire* (*i.e.* the intention to retire as soon as possible) is the outcome variable of a binary nature, with ‘No’ coded 0 and ‘Yes’ coded 1. These data have been collected in the employment module via the question ‘Thinking about your present job, would you like to retire as early as you can from this job?’.

### Explanatory variables

The main predictors in the analysis indicate digital/ICT/computer skills and family structure. The *digital skills* were measured on a 6-point scale from ‘Never used a computer’ to ‘Excellent’ by asking ‘How would you rate your computer skills? Would you say they are…’. Then, the *demand for digital skills at work* is indicated by the binary response (No = 0, Yes = 1) to the question ‘Does your current job require using a computer?’ Four variables indicate the family structure; a *partner* in the household (No = 0, Yes = 1), *number of living parents*, *number of children* (with the highest category 4 and more), *and number of grandchildren* (with the highest category 4 and more). Moreover, the regression models use other important explanatory and control variables. These characteristics are *age*, *gender* (Men = 0, Women = 1), *level of education* (ISCED 0, 1 = 1, ISCED 2–4 = 2, ISCED 5, 6 = 3), *type of job* based on ISCO (Low skilled blue collar = 1; High skilled blue collar = 2; Low skilled white collar = 3; High skilled white collar = 4), *type of employer* (Private sector = 1, Public sector = 2, Self-employed = 3), subjective *financial situation* (Poor = 1, Good = 2, Very good = 3), subjective *health status* (Poor or fair = 1, Good = 2, Very good or excellent = 3), and *wave* of data collection (Wave 5 = 1, Wave 6 = 2, Wave 7 = 3). Table 4 in the Supplementary material provides more details on the construction of variables.

### Analytical strategy

This study compares two analytical strategies for panel data structure—random-effect and fixed-effect regression—applied to an identical final sample. The random-effect model (REM) uses both between-person variation and within-person variation for its estimation. Hence, REM works on principles similar to standard regression models and provides almost identical results. In contrast, the fixed-effect model (FEM) uses only the within-person variation (not the between-person variation) to evaluate changes within individuals and their effect over time (Allison 2009). In this sense, FEM makes it possible to use every individual as their control, thus controlling all stable (un)observable macro- and micro-characteristics. This method’s robustness is conditioned by a sufficient variation of predictors and their sufficient exogeneity (Windmeijer 2000), with higher standard errors in fixed-effects models indicating a low efficiency of estimation (Allison 2009). Apart from low statistical power, FEM can also suffer from an inability to include time-invariant variables, unclear theoretical contribution, and other possible drawbacks (Hill et al. 2020). FEM is preferable to REM if they differ, as the difference indicates biased REM estimates. While the analysis aims for robust and potentially causal findings, the explanatory variables (e.g. digital skills) are not strictly exogenous, and thus the REM/FEM difference rather tests the random effects assumption than shows indisputable causal effects (Allison 2009; Brüderl and Ludwig 2015).

An alternative to comparing REM and FEM is the within-between RE model (WBREM), often labelled as a hybrid. WBREM combines the utilities of REM and FEM, including their strengths, being more flexible in several aspects (Dieleman and Templin 2014; Bell et al. 2019). However, WBREM has a drawback of higher complexity, presenting RE, within-estimators, and between-estimators in one model; the WBREM models are presented in Table 5 in the Supplementary material. The paper compares REM with FEM, which is the most suitable procedure for its research aim of presenting results parallel with cross-sectional analyses and their differences from changes within individuals. This procedure assesses the within-person effect of changes (through training and individual development) of digital skills or changes in family composition on retirement intentions. After the REM-FEM comparison, the more suitable model is estimated for men and women separately to evaluate possible gender differences derived from the literature. The analysis estimates conditional logit models using Stata 16.1 with commands *xtlogit* (for REM and FEM) and *xthybrid* (for WBREM). Additionally, the same models were estimated by *xtreg* command as FE linear regression models to provide the possibility of approximating average marginal effects (Tables 6 and 7 in the Supplementary material). The minor differences between REM/FEM and WBREM coefficients are attributable to a different estimation function (conditional logit vs generalised linear model using a logit link function).

## Results

### REM and FEM differences

The first model for REM in Table 2 shows the odds ratios (OR) and standard errors of predictors if both between- and within-person variation are used. Better ITC skills prevent retirement intentions with approximately linear coefficients; for instance, older workers with excellent ITC skills have 35% lower odds of retirement intentions. The coefficient for digital skills holds even when job type and job satisfaction is in the model.^1^ In contrast to this notable relationship, a binary indicator for the demand for digital skills is negligible in the model. The second area of interest is the family structure, which has significant coefficients for three out of four indicators. Specifically, having a partner increases the odds of retirement decision by 24%; having more children decreases the odds and having more grandchildren increases them. The interpretation would be that grandchildren create caregiving demand, children increase motivation for higher earnings and partner motivation for spending joint time at home. The decision to retire increases with age and appears more often among men (women have 0.82* lower odds). Then, working in the private vs public sector does not make a difference, but being self-employed lowers the odds of retirement intentions by 64% (although this coefficient may be partly attributed to different retirement meanings and options for self-employed). The coefficient for a wave of data collection only says that the decision to retire is more frequent in later waves. Finally, better health, higher education, a better financial situation, and high skilled white-collar jobs prevent retirement intentions.

**Table 2:** Random-effects and fixed-effects regression predicting the decision to retire *Source:* These calculations use data from SHARE, Waves 5, 6, and 7

	Random-effects model (REM)		Fixed-effects model (FEM)	
	OR	SE	OR	SE
Digital skills (Never used a computer is the ref.)				
Poor	1.085	0.155	1.148	0.230
Fair	0.879	0.124	0.979	0.212
Good	0.753 +	0.110	0.961	0.218
Very good	0.690*	0.108	1.010	0.245
Excellent	0.647*	0.111	0.986	0.263
Demand for digital skills at work (yes)	1.086	0.090	0.943	0.118
Partner in household (yes)	1.240**	0.101	1.502 +	0.352
Number of parents (0 is the ref.)				
1	1.034	0.069	0.866	0.133
2	1.080	0.100	0.843	0.205
Number of children (0 is the ref.)				
1	1.039	0.128	1.187	0.344
2	0.876	0.101	1.157	0.327
3	0.790 +	0.102	1.107	0.358
4 and more	0.723*	0.109	0.921	0.330
Number of grandchildren (0 is the ref.)				
1	1.498***	0.129	1.583**	0.225
2	1.577***	0.146	1.695**	0.288
3	1.620***	0.187	1.436 +	0.297
4 and more	1.465***	0.154	1.091	0.227
Employer (Private sector is the ref.)				
Public sector	1.133 +	0.077	0.846	0.110
Self-employed	0.363***	0.034	0.678*	0.124
Health (Poor or fair is the ref.)				
Good	0.539***	0.041	0.725**	0.075
Very good or excellent	0.337***	0.028	0.664**	0.080
Financial situation (Poor is the ref.)				
Good	0.774**	0.058	0.927	0.092
Very good	0.720***	0.057	0.898	0.100
Wave (Wave 5 is the ref.)				
Wave 6	1.572***	0.074	1.354***	0.063
Wave 7	2.238***	0.269	1.678***	0.222
Age	2.634***	0.526		
Age squared	0.991***	0.002		
Gender (Woman)	0.822**	0.056		
Education (ISCED 0, 1 is the ref.)				
ISCED 2–4	1.054	0.148		
ISCED 5, 6	0.801	0.124		
Type of job (Low skilled blue collar is the ref.)				
High skilled blue collar	0.762*	0.096		
Low skilled white collar	0.640***	0.070		
High skilled white collar	0.464***	0.055		
Country (14 dummies)	Not shown	Not shown		
*N*—measures in time	18,940		18,940	
*N*—respondents	9126		9126	

Significance levels +*p* < 0.1, *p < 0.05, **p < 0.01, ***p < 0.001

Table 2 also presents estimates for FEM. This model does not include the variables gender and country (both stable across time), level of education (negligible number of changes), and age (constant changes over time). Rather than by age, the time dimension is represented by wave, which indicates an increase in retirement intentions among waves of data collection. Since every individual makes their own control, omitting some variables does not pose a problem. Regarding the coefficients (indicating the effect of change over time), changes in ITC skills (or in demand for them) do not affect the odds of retirement decision (H1a and H1b rejected).^2^ The coefficient of a partner in the households has increased compared to REM (and higher *p* = 0.083 results from the lower efficiency of estimates). Thus, losing a partner decreases the odds of retirement intentions by 50% (or getting a partner increases the odds). The number of children does not affect retirement intentions, but the number of grandchildren still reduces them in FEM (H2a and H2b hold, H2c not). While a change in a financial situation does not have any effect, improving health status and starting to work as self-employed prevent retirement intentions (H3a and H3b hold, but H3c does not).

Clearly, many REM and FEM coefficients differ. This difference is supported by the Hausman test (*p* < 0.00001) and comparison of within- and between-effects in WBREM (Table 5 in the Supplementary material). These findings indicate that the random effects assumption does not hold and individual changes relate to the retirement decision differently than differences among individuals. While RE coefficients are considered spurious, FEM preferred in this panel analysis suffers from lower efficiency indicated by higher standard errors (SE). As mentioned above, the coefficient of a partner in the household on the borderline of statistical significance (and a strong effect of becoming self-employed with mildly significant *p* = 0.033) suggests a lower statistical power of FEM. Still, the paper argues that the most notable differences between the models (ITC skills and financial situation) differ in their coefficients, the number of changes is adequate, and previous associations could be explained by some unobserved variables. For instance, the coefficients for digital skills became negligible, although this variable evinces the largest number of changes—4900. The only variables with fewer than 1000 changes are partnership (303) and number of children (947). Therefore, the variable with much fewer changes than any other has a coefficient on the borderline of statistical significance, showing the statistical power for others can be sufficient.

### Gender differences in FEM

The next part of the analysis builds on the higher robustness of FEM estimation and tests gender differences in predictors of retirement intentions (H4a-H4c). Table 3 shows FEM for men and women separately, with most effects similar to FEM for the pooled sample (Table 2). Namely, the impact of digital skills and digital demands is significant neither for men nor for women. The OR are around 1 for the categories of digital skills compared to workers with no computer experience. The coefficient for women with excellent digital skills is 1.26, but even this OR has a *p* = 0.567.

**Table 3:** Fixed-effects regression predicting the decision to retire separately for men and women. *Source:* These calculations use data from SHARE, Waves 5, 6, and 7

	FEM: men		FEM: women	
	OR	SE	OR	SE
Digital skills (Never used a computer is the ref.)				
Poor	1.375	0.373	1.043	0.319
Fair	1.020	0.301	1.028	0.336
Good	1.176	0.368	0.903	0.307
Very good	1.203	0.406	0.969	0.350
Excellent	0.865	0.323	1.255	0.497
Demand for digital skills at work (yes)	1.130	0.197	1.043	0.319
Partner in household (yes)	1.935 +	0.700	1.288	0.410
Number of parents (0 is the ref.)				
1	1.074	0.245	0.736	0.157
2	1.121	0.404	0.703	0.240
Number of children (0 is the ref.)				
1	0.598	0.234	2.975*	1.528
2	0.943	0.363	1.933	0.932
3	1.601	0.710	1.278	0.705
4 and more	0.871	0.436	1.342	0.783
Number of grandchildren (0 is the ref.)				
1	1.250	0.272	1.900**	0.370
2	1.067	0.268	2.299**	0.553
3	0.835	0.260	2.009*	0.578
4 and more	0.838	0.252	1.277	0.384
Employer (Private sector is the ref.)				
Public sector	0.852	0.181	0.876	0.148
Self-employed	0.774	0.173	0.536 +	0.175
Health (Poor or fair is the ref.)				
Good	0.726*	0.111	0.717*	0.102
Very good or excellent	0.675*	0.119	0.631**	0.107
Financial situation (Poor is the ref.)				
Good	0.936	0.137	0.925	0.129
Very good	0.859	0.139	0.931	0.148
Wave (Wave 5 is the ref.)				
Wave 6	1.236**	0.084	1.461***	0.094
Wave 7	1.626*	0.316	1.750**	0.322
*N*—measures in time	8908		10,032	
*N*—respondents	4298		4828	

Significance levels +*p* < 0.1, *p < 0.05, **p < 0.01, ***p < 0.001

The role of family structure differs by gender substantially. Getting a new partner is more important for men; the odds of planning to retire increase by 94%. In contrast, retirement intentions strongly increase for women getting their first child (this happened through re-partnering and involves only 12 cases) and women with additional grandchild(ren). The effects on the borderline of significance seem plausible amid a lower efficiency of split-sample FEM models and their strong, substantial meaning. Then, both men and women evince no significant effect of changing the number of living parents and financial situation on retirement intentions; the negative effect of health improvement from REM holds for both sexes. The previously identified effect of becoming self-employed on postponing retirement occurs only in women. Specifically, older female workers becoming self-employed have a 46% lower chance of intending to retire (*p* = 0.056), while men in the same situation have a 23% lower chance (*p* = 0.251). It is crucial to realise that these robust findings bring a unique contribution, but the statistical power of these models is low and further replication is needed. At this stage, the paper illustrates the differences between the within-person estimation and the one used by most studies and sketches the implications in the concluding section.

## Conclusions and discussion

The present study explores *several types of possible causal factors affecting the retirement decision-making of older European workers*, with particular attention to the gender differences in these factors. It capitalises on the panel dimension of SHARE data to observe changes within individuals, comparing this analytical approach to a standard type of regression analysis. Several types of predictors of retirement intentions are tested, including those studied more often (health, financial situation) and less frequently (digital skills, family structure, type of employer). Finally, the study accompanies findings for a pooled sample by a gender-specific analysis of theoretically grounded expectations.

The coefficients based on REM show a positive relationship between digital skills, number of children, being self-employed, health, financial situation, and being a woman and planning to work longer. In contrast, having a partner and more grandchildren was associated with intentions to retire as soon as possible. These relationships were consistent with most of the previous (cross-sectional) studies (Hochman and Lewin-Epstein 2013; Radl 2013; Dingemans et al. 2017; Axelrad and Mcnamara 2018; Pilipiec et al. 2022) but substantially differed from within-person effects provided by FEM. Based on the Hausman test and WBREM, the random effects assumption is not fulfilled, and *more robust FEM results not present in previous research* constitute the main findings of the study. It is worth noticing that FEM provides findings with a different meaning and interpretation, as illustrated below.

First, *change in digital skills does not affect retirement intentions*, even though the data provide enough changes within individuals. While the role of binary-measured demand for digital skills at the current job was significant neither in REM nor in FEM and may be accounted for by a weak indicator and/or agency of older workers, the coefficients for digital skills are much more surprising. This 6-point self-evaluation scale has revealed no difference between any of its categories. While these findings are relatively robust to various model specifications, it is vital to remember that the models and effects are not strictly exogenous. In any case, the argument that further ICT training of older adults could extend their working life (Lee et al. 2008) remains unsupported by any study. This relationship has severe implications for the employability of older adults if supported by future research, although it could end up differently, for instance, in experimental design involving highly motivated groups. Intense training aimed at some positions may help older workers to get/keep the job. However, the general empirical pattern is not strong (and could be accounted for by other unobserved factors, such as work opportunities or motivation). The theoretical aspect is also important; *the results do not show any inception of digital capital in Bourdieu’s sense* (Bourdieu 1984) as a new type of source shaping and reproducing inequalities in the labour market.

Second, the only indicators of family structure related to retirement intentions are *partnership and more grandchildren, both connected to a higher propensity for retirement plans*. The effect of a partner in the household was strong despite a low number of changes in this variable and can be interpreted either as a gain of the partner increasing the retirement intentions or a loss of the partner decreasing them. While having fewer children was related to higher retirement intentions in REM but not in FEM, the same effect of more grandchildren remained. Hence, *the number of children and living parents is not connected to retirement intentions*, *but the number of grandchildren is*. More interestingly, the relationship between additional grandchildren and higher retirement intentions was identified only in the subsample of women, who seem to adjust their career plans much more often. This conclusion goes against the previous quantitative studies indicating the non-existent association between caregiving and retirement (Szinovacz et al. 2001; Forma 2009; Dingemans et al. 2017) and a weak-to-none role of having children and grandchildren (Hochman and Lewin-Epstein 2013; Radl 2013; Dingemans et al. 2017). An option for future research is examining the effect of family/caregiving on actual retirement behaviour, which is not possible in the presented type of analysis.

Third, self-employment and better health lead to a higher willingness to work longer, while changes in the subjective financial situation have no within-person effect. Being self-employed could be connected to lower pension eligibility when reaching pension age, as Wahrendorf et al. (2017) suggest. However, the possible causal effect of becoming self-employed rather supports the argument that *more flexible settings of being self-employed could constitute a relevant bridge job for some older workers* (Lain and Vickerstaff 2014), although both these mechanisms can be at play. Self-employment decreases retirement intentions for women more than men, which could be interpreted by gendered ageism (Krekula et al. 2018) being stronger in regular jobs. The strong detrimental effect of worsening health status on higher retirement intentions documented by previous research (Fisher et al. 2016; Dingemans et al. 2017; Wahrendorf et al. 2017) is also supported by these findings. Finally, the association between financial situation and willingness to work longer from REM does not appear in FEM. This difference means that the relationship appearing in the cross-sectional studies (Pilipiec et al. 2022) seems spurious.

It is crucial to keep in mind that these findings bring a unique contribution. Still, the statistical power of these models is low, and further replication with larger samples and other methods—including qualitative research—is needed. Then, the categorical and self-evaluated indicator of digital skills is far from being a perfect measurement of the concept. This regressor contains a measurement error, and the potential attenuation bias can increase when a between-person variability is not used (Ashenfelter and Krueger 1994); more research with other types of data is needed. Moreover, *this study does not deal with the country-specific pension and social policy systems, individual pension eligibility, and actual exit from the labour market*, which are aspects addressed by the previous studies (Dingemans et al. 2017; Axelrad and Mcnamara 2018; Dudová and Pospíšilová 2022). At this stage, the paper illustrates the differences between the within-person estimator and REM as a representative of common types of estimations prone to provide spurious effects. Despite several limitations in terms of data, indicators (especially measurement of digital skills and their demand), and analytical techniques, this paper provides an essential contribution to retirement decision-making by showing that some crucial factors of previous studies do not hold in panel data or work gender-specifically.

### Supplementary Information

Below is the link to the electronic supplementary material.

Supplementary file1 (PDF 743 kb)

## Footnotes

1 The role of digital skills is also substantially the same with other controls (such as job satisfaction) included. However, job satisfaction is one of the variables omitted from the final models, because a) only 7.5% of working respondents (longitudinal respondents with a job change) provided this information in Wave 5, b) addressing a large number of missing values by coping values from all subsequent and consequent waves is controversial, c) very low number of changes make this variable available only for REM, and d) a resulting sample reduction was problematic, especially for FEM models based on the same sample.

2 Additional sensitivity analysis estimated the models with different categorisations and different reference categories. The linear effect of digital skills in REM (or cross-sectional models) holds, while there is no hint of difference among any categories of this variable in FEM.
