# Supplementary material for: Effects of digital skills and other individual factors on retirement decision-making and their gender differences
Source: Eur J Ageing. 2023 Oct 7;20(1):38. doi: 10.1007/s10433-023-00784-9 (PMC10560239; doi:10.1007/s10433-023-00784-9)
Supplement: Supplementary file 1 — Supplementary file1 (PDF 743 kb) [file 10433_2023_784_MOESM1_ESM.pdf]

Table 4: Construction and definition of more complex variables

Variable	Question	Categories – original and analysed
**Decision to retire**	Thinking about your present job, would you like to retire as early as you can from this job?	1. Yes=1<br>5. No=0
**Digital skills**	How would you rate your computer skills? Would you say they are...	1. Excellent → Excellent=6<br>2. Very good → Very good=5<br>3. Good → Good=4<br>4. Fair → Fair=3<br>5. Poor → Poor=2<br>6. I never used a computer (SPONTANEOUS ONLY) → Never used a computer=1
**Demand for digital skills at work**	Does your current job require using a computer?	1. Yes=1<br>5. No=0
**Employer**	In this job were you a private-sector employee, a public sector employee or self-employed?	1. Private sector employee → Private sector=1<br>2. Public sector employee → Public sector=2<br>3. Self-employed → Self-employed=3
**Health**	Would you say your health is...	1. Excellent → Very good or excellent=3<br>2. Very good → Very good or excellent=3<br>3. Good → Good=2<br>4. Fair → Poor or fair=1<br>5. Poor → Poor or fair=1
**Financial situation**	Thinking of your household's total monthly income, would you say that your household is able to make ends meet...	1. With great difficulty → Poor=1<br>2. With some difficulty → Poor=1<br>3. Fairly easily → Good=2<br>4. Easily → Very good=3
**Type of job**	Please look at card {SHOWCARD_ID}. What best describes this job?	ISCO codes 1000-3999 → High skilled white collar=4<br>ISCO codes 4000-5999 → Low skilled white collar=3<br>ISCO codes 6000-7999 → High skilled blue collar=2<br>ISCO codes 8000-9999 → Low skilled blue collar=1<br>ISCO codes 1000 and more → dropped

Table 5: Models of the within-between RE model (WBREM)

	WBREM for a pooled sample		WBREM: men		WBREM: women	
	**OR**	**SE**	**OR**	**SE**	**OR**	**SE**
**Random effects**						
**Age**	6.754***	1.834	5.373***	2.172	7.047***	2.635
**Age squared**	0.982***	0.002	0.984***	0.004	0.982***	0.003
**Gender** (Woman)	0.821**	0.058	omitted		omitted	
**Education** (ISCED 0, 1 is the ref.)						
ISCED 2-4	1.154	0.172	1.495+	0.317	0.869	0.184
ISCED 5, 6	0.917	0.150	1.036	0.245	0.740	0.172
**Type of job** (Low skilled blue collar is the ref.)						
High skilled blue collar	0.794+	0.107	0.765	0.131	0.769	0.185
Low skilled white collar	0.666**	0.078	0.732+	0.126	0.653*	0.109
High skilled white collar	0.509***	0.065	0.584**	0.103	0.477***	0.089
**Country** (14 dummies)	Not shown		Not shown		Not shown	
**Within-estimators**						
**Digital skills** (Never used a computer is the ref.)						
Poor	1.192	0.250	1.638	0.501	0.942	0.275
Fair	1.002	0.225	1.157	0.377	0.937	0.296
Good	1.003	0.237	1.387	0.476	0.813	0.269
Very good	1.042	0.262	1.384	0.508	0.873	0.306
Excellent	0.963	0.267	0.995	0.396	1.035	0.406
**Demand for digital skills at work** (yes)	0.903	0.118	1.116	0.207	0.717+	0.134
**Partner in household (yes)**	1.550+	0.371	2.047*	0.721	1.301	0.430
**Number of parents** (0 is the ref.)						
1	0.840	0.134	1.085	0.258	0.699	0.152
2	0.797	0.203	1.153	0.442	0.614	0.212
**Number of children** (0 is the ref.)						
1	1.190	0.374	0.549	0.235	2.903*	1.384
2	1.153	0.352	0.811	0.347	1.856	0.830
3	1.052	0.361	1.362	0.646	0.977	0.499
4 and more	0.936	0.348	0.713	0.377	1.371	0.735
**Number of grandchildren** (0 is the ref.)						
1	1.537**	0.212	1.251	0.261	1.837**	0.343
2	1.714**	0.293	1.153	0.294	2.371***	0.554
3	1.433+	0.297	0.882	0.278	2.104**	0.587
4 and more	1.030	0.227	0.840	0.278	1.224	0.366
**Employer** (Private sector is the ref.)						
Public sector	0.816	0.112	0.852	0.191	0.812	0.142
Self-employed	0.684+	0.134	0.781	0.195	0.568+	0.183
**Health** (Poor or fair is the ref.)						
Good	0.695**	0.074	0.723*	0.113	0.666**	0.096
Very good or excellent	0.640***	0.079	0.651*	0.117	0.622**	0.106
**Financial situation** (Poor is the ref.)						
Good	0.941	0.096	0.925	0.139	0.946	0.134

Very good	0.925	0.106	0.857	0.143	0.980	0.156
**Wave** (Wave 5 is the ref.)						
Wave 6	1.333***	0.061	1.231**	0.083	1.432***	0.091
Wave 7	1.885***	0.262	1.854**	0.396	1.919***	0.357
**Between-estimators**						
**Digital skills** (Never used a computer is the ref.)						
Poor	1.149	0.247	1.248	0.378	1.041	0.323
Fair	0.899	0.174	1.082	0.297	0.783	0.219
Good	0.685+	0.138	0.653	0.187	0.674	0.195
Very good	0.574*	0.125	0.593+	0.184	0.512*	0.159
Excellent	0.586*	0.138	0.475*	0.156	0.723	0.252
**Demand for digital skills at work** (yes)	1.275*	0.147	1.142	0.197	1.333+	0.210
**Partner in household (yes)**	1.264***	0.112	1.137	0.170	1.340**	0.151
**Number of parents** (0 is the ref.)						
1	1.089	0.082	1.086	0.122	1.090	0.112
2	1.169	0.119	1.030	0.158	1.285+	0.175
**Number of children** (0 is the ref.)						
1	1.061	0.145	1.519*	0.313	0.792	0.148
2	0.839	0.106	1.071	0.202	0.684*	0.120
3	0.754*	0.108	0.941	0.199	0.618*	0.122
4 and more	0.675*	0.114	0.922	0.225	0.533**	0.127
**Number of grandchildren** (0 is the ref.)						
1	1.555***	0.186	1.665**	0.305	1.481*	0.236
2	1.455**	0.171	1.172	0.211	1.742***	0.273
3	1.696**	0.262	1.311	0.317	2.013**	0.406
4 and more	1.695***	0.213	2.035***	0.393	1.471*	0.246
**Employer** (Private sector is the ref.)						
Public sector	1.198*	0.096	1.059	0.135	1.214+	0.129
Self-employed	0.306***	0.034	0.330***	0.048	0.262***	0.048
**Health** (Poor or fair is the ref.)						
Good	0.475***	0.055	0.446***	0.077	0.493***	0.077
Very good or excellent	0.207***	0.024	0.201***	0.036	0.207***	0.033
**Financial situation** (Poor is the ref.)						
Good	0.713**	0.083	0.781	0.138	0.662**	0.103
Very good	0.647***	0.072	0.736+	0.126	0.580***	0.087
**Wave** (Wave 5 is the ref.)						
Wave 6	0.697	1.051	0.423	0.964	0.904	1.828
Wave 7	0.242	0.232	0.097	0.143	0.513	0.658
**N – measures in time**	**18,940**		**8,908**		**10,032**	
**N – respondents**	**9,126**		**4,298**		**4,828**	

Source: These calculations use data from SHARE, Waves 5, 6, and 7.
Significance levels: + p<0.1, * p<0.05, ** p<0.01, *** p<0.001.

Table 6: Random-effects and fixed-effects linear regression for approximating average marginal effects

	Random-effects model (REM)		Fixed-effects model (FEM)	
	**Coef.**	**SE**	**Coef.**	**SE**
**Digital skills** (Never used a computer is the ref.)				
Poor	0.012	0.018	0.016	0.026
Fair	-0.016	0.018	-0.005	0.028
Good	-0.035+	0.019	-0.005	0.029
Very good	-0.047*	0.020	0.000	0.031
Excellent	-0.055*	0.022	-0.008	0.035
**Demand for digital skills at work** (yes)	0.010	0.011	-0.009	0.016
**Partner in household (yes)**	0.027*	0.010	0.050+	0.030
**Number of parents** (0 is the ref.)				
1	0.005	0.008	-0.023	0.020
2	0.011	0.012	-0.032	0.032
**Number of children** (0 is the ref.)				
1	0.005	0.016	0.023	0.041
2	-0.017	0.015	0.028	0.039
3	-0.031+	0.017	0.016	0.044
4 and more	-0.041*	0.019	0.003	0.047
**Number of grandchildren** (0 is the ref.)				
1	0.052***	0.011	0.049**	0.017
2	0.058***	0.012	0.064**	0.021
3	0.062***	0.015	0.043+	0.026
4 and more	0.049***	0.013	0.007	0.027
**Employer** (Private sector is the ref.)				
Public sector	0.015+	0.009	-0.025	0.017
Self-employed	-0.126***	0.012	-0.055*	0.024
**Health** (Poor or fair is the ref.)				
Good	-0.081***	0.010	-0.049***	0.013
Very good or excellent	-0.141***	0.011	-0.058***	0.016
**Financial situation** (Poor is the ref.)				
Good	-0.032**	0.010	-0.007	0.013
Very good	-0.041**	0.010	-0.011	0.014
**Wave** (Wave 5 is the ref.)				
Wave 6	0.058***	0.006	0.037**	0.006
Wave 7	0.103***	0.015	0.055**	0.015
**Age**	0.116***	0.025		
**Age squared**	-0.001***	0.000		
**Gender** (Woman)	-0.025**	0.009		
**Education** (ISCED 0, 1 is the ref.)				
ISCED 2-4	0.005	0.018		
ISCED 5, 6	-0.030	0.020		
**Type of job** (Low skilled blue collar is the ref.)				
High skilled blue collar	-0.037*	0.016		
Low skilled white collar	-0.059***	0.014		
High skilled white collar	-0.099***	0.015		

**Country** (14 dummies)	Not shown	
**N – measures in time**	**18,940**	**18,940**
**N – respondents**	**9,126**	**9,126**

Source: These calculations use data from SHARE, Waves 5, 6, and 7.
Significance levels: + p<0.1, * p<0.05, ** p<0.01, *** p<0.001.

Table 7: Fixed-effects linear regression for approximating average marginal effects for men and women

	**FEM: men**		**FEM: women**	
	**Coef.**	**SE**	**Coef.**	**SE**
**Digital skills** (Never used a computer is the ref.)				
Poor	0.044	0.038	-0.005	0.035
Fair	0.005	0.040	-0.007	0.039
Good	0.024	0.042	-0.024	0.041
Very good	0.031	0.045	-0.020	0.043
Excellent	-0.009	0.049	0.004	0.049
**Demand for digital skills at work** (yes)	0.016	0.023	-0.037	0.024
**Partner in household (yes)**	0.079+	0.043	0.032	0.042
**Number of parents** (0 is the ref.)				
1	0.005	0.028	-0.043	0.027
2	0.004	0.047	-0.055	0.043
**Number of children** (0 is the ref.)				
1	-0.081	0.055	0.142*	0.060
2	-0.026	0.055	0.088	0.056
3	0.036	0.060	0.006	0.064
4 and more	-0.041	0.066	0.050	0.067
**Number of grandchildren** (0 is the ref.)				
1	0.022	0.025	0.071**	0.023
2	0.012	0.031	0.110***	0.029
3	-0.017	0.038	0.097**	0.035
4 and more	-0.022	0.040	0.034	0.038
**Employer** (Private sector is the ref.)				
Public sector	-0.015	0.028	-0.027	0.022
Self-employed	-0.041	0.031	-0.073+	0.039
**Health** (Poor or fair is the ref.)				
Good	-0.044*	0.019	-0.055**	0.018
Very good or excellent	-0.054*	0.022	-0.062**	0.022
**Financial situation** (Poor is the ref.)				
Good	-0.009	0.019	-0.007	0.018
Very good	-0.019	0.021	-0.002	0.020
**Wave** (Wave 5 is the ref.)				
Wave 6	0.026**	0.008	0.047***	0.008
Wave 7	0.053*	0.021	0.056**	0.021
**N – measures in time**	**8,908**		**10,032**	
**N – respondents**	**4,298**		**4,828**	

Source: These calculations use data from SHARE, Waves 5, 6, and 7.
Significance levels: + p<0.1, * p<0.05, ** p<0.01, *** p<0.001.
